# Glycine Protects Muscle Cells From Wasting *in vitro* via mTORC1 Signaling

**DOI:** 10.3389/fnut.2019.00172

**Published:** 2019-11-13

**Authors:** Marissa K. Caldow, Daniel J. Ham, Jennifer Trieu, Jin Dylan Chung, Gordon S. Lynch, René Koopman

**Affiliations:** Centre for Muscle Research, Department of Physiology, The University of Melbourne, Melbourne, VIC, Australia

**Keywords:** atrophy, amino acids, muscle wasting, starvation, C2C12, protein synthesis

## Abstract

Glycine supplementation can protect skeletal muscles of mice from cancer-induced wasting, but the mechanisms underlying this protection are not well-understood. The aim of this study was to determine whether exogenous glycine directly protects skeletal muscle cells from wasting. C2C12 muscle cells were exposed to non-inflammatory catabolic stimuli via two models: serum withdrawal (SF) for 48 h; or incubation in HEPES buffered saline (HBS) for up to 5 h. Cells were supplemented with glycine or equimolar concentrations of L-alanine. SF- and HBS-treated myotubes (with or without L-alanine) were ~20% and ~30% smaller than control myotubes. Glycine-treated myotubes were up to 20% larger (*P* < 0.01) compared to cells treated with L-alanine in both models of muscle cell atrophy. The mTORC1 inhibitor rapamycin prevented the glycine-stimulated protection of myotube diameter, and glycine-stimulated S6 phosphorylation, suggesting that mTORC1 signaling may be necessary for glycine's protective effects *in vitro*. Increasing glycine availability may be beneficial for muscle wasting conditions associated with inadequate nutrient intake.

## Introduction

The non-essential amino acid glycine is often considered biologically neutral, and not required for the regulation of protein synthesis under normal healthy conditions. However, reduced intracellular levels of glycine have been reported in older individuals (1) and in mouse models of diabetes and muscular dystrophy (2, 3), suggesting that either glycine metabolism is increased during these conditions, or tissue demand exceeds dietary intake. Our observations in several mouse models of muscle wasting showed that supplementation with glycine preserved muscle mass and metabolic function in a range of conditions where the anabolic response to nutrition was altered (4–6). Glycine administration attenuated skeletal muscle wasting and loss of physical function in a mouse model of cancer cachexia, which was associated with a reduction in protein breakdown and skeletal muscle markers of inflammation and ROS (4). In a mouse model of acute (LPS-induced) inflammation, glycine administration preserved the skeletal muscle anabolic response to leucine, through upregulation of mTORC1 signaling and preservation of protein synthesis. Glycine can affect cell homeostasis via glycine receptor mediated signaling and via its metabolism (7). Indeed, previous reports have linked glycine receptor-mediated signaling, via its scaffolding protein gephyrin, to mTORC1 activation in other tissues (8). In the *in vivo* LPS model we also showed a reduction in oxidative stress (DHE) but not mRNA expression of pro-inflammatory cytokines and chemokines in skeletal muscle (6). Dietary glycine supplementation in a mouse model of caloric restriction reduced adiposity (whole-body and epididymal fat mass) and preserved lean mass and muscle mass (5). Together, these data revealed a positive effect of glycine treatment on skeletal muscle protein metabolism, mass and function during muscle wasting conditions. However, it is currently unclear whether the beneficial effects of glycine on skeletal muscle are entirely the result of inflammatory cell inactivation, or whether glycine has muscle cell-specific effects. We tested the hypothesis that glycine would directly attenuate myotube wasting in an mTORC1-dependent manner.

We aimed to determine whether exogenous glycine protects muscle cells from cachectic stimuli. To investigate the effect of glycine on myotube wasting mature C2C12 myotubes were supplemented with glycine or equimolar concentrations of L-alanine and atrophy induced via 2 different approaches: serum withdrawal for 48 h; or incubation in HEPES buffered saline for up to 5 h.

## Methods

### Cell Culture

Murine C2C12 myoblasts (Cryosite distribution, NSW, Australia) were cultured in DMEM (Life Technologies, Australia) containing 10% (v/v) fetal calf serum (Life Technologies), 1% L-glutamine (v/v) (Life Technologies), and 1% (v/v) antibiotic solution (100 unit/ml penicillin/streptomycin, Life Technologies) at 37°C in an atmosphere of 5% CO_2_. Upon confluency, the media was changed to differentiation media [DMEM containing 2% (v/v) horse serum, 1% L-glutamine and 1% antibiotic solution (Life Technologies)] for 5 days to promote formation of mature multinucleated myotubes (9).

#### Wasting Conditions

To induce wasting via growth factor deprivation, cells were washed once in serum free DMEM (Life Technologies, Australia) and then incubated in DMEM (i.e., standard amino acid composition) containing 1% L-glutamine and 1% antibiotic solution (Life Technologies) but lacking 2% horse serum for 48 h (SF) (9). SF was supplemented with an additional 2.5 mM glycine (Sigma-Aldrich, Castle Hill, NSW, Australia) or L-alanine (Sigma-Aldrich). To induce wasting via nutrient starvation, cells were washed once in HEPES buffered saline (HBS; 20 mM HEPES/Na pH 7.4, 140 mM NaCl, 2.5 mM MgSO_4_, 5 mM KCl, and 1 mM CaCl_2_, no amino acids present), then incubated in HBS (9, 10) with glycine or equimolar concentrations of L-alanine for up to 5 h. L-alanine serves as an isonitrogenous control as it does not modulate cell size and protein turnover in cell and animal models (4–6, 9, 10).

Rapamycin (100 nM, Sigma-Aldrich) was used to inhibit mTORC1 activation (10). We have previously reported that these atrophy models are not associated with altered myotube viability as assessed by Trypan Blue staining (9).

#### Glycine Withdrawal

DMEM media was formulated without glycine (Life Technologies). Basal levels (0.4 mM) or additional amounts (2.5 mM) of glycine (Sigma-Aldrich) were added when appropriate to serum free or differentiation media, as specified.

### Myotube Diameter

Cells were washed 2 × 5 min in phosphate buffered saline (PBS) and then fixed with 4% paraformaldehyde/PBS for 15 min. Cells were then washed in PBS (3 × 5 min), permeated with 0.1% TritonX-100/PBS, washed in PBS (3 × 5 min) and then incubated in 3% bovine serum albumin (BSA)/PBS for 2 h. Cells were incubated with primary antibody overnight at 4°C. MF20 (1:50; Developmental Studies Hybridoma Bank, University of Iowa, Department of Biology, Iowa City, IA, USA) in 3% BSA/PBS was used to stain myosin heavy chain (MHC). Cells were then washed with PBS (3 × 5 min) and incubated in goat-anti-mouse IgG2b Alexa555 secondary antibody (1:500, Life Technologies) and DAPI (1:1,000) for 2 h in 3% BSA/PBS. Cells were washed in PBS (3 × 5 min) and then imaged on a Zeiss Axiovert 40 CFL inverted microscope using a 10X objective. Four images were taken in each well from pre-defined locations within each quadrant. Myotube diameter was measured using AxioVision software (AxioVision AC Rel. 4.8.2, Carl Zeiss Imaging Solutions, Wrek, Göttingen; Germany). A total of ~50–80 myotubes were measured per well and the average diameter of each well was used for statistical analysis as described previously (6, 9, 10).

### Protein Synthesis

Myotubes were grown and treated as described previously. To determine the rate of protein synthesis we utilized SUnSET methodology, as described (10, 11). Briefly, puromycin (Sigma-Aldrich) was administered to the media at a final concentration of 1 μM exactly 30 min before cells were collected in ice-cold homogenizing buffer. Anti-puromycin was purchased from Millipore (Kilsyth, Victoria, Australia) and immunoblotting was used to detect changes in puromycin incorporation as described (9, 10).

### Protein Extraction and Immunoblotting

Cell lysates were prepared using RIPA lysis buffer (Merck Millipore, VIC, Australia) including protease and phosphatase inhibitor cocktails (Sigma-Aldrich). Lysates were rotated end over end at 4°C for 30 min, then centrifuged at 13,000 RPM at 4°C for 10 min to collect the supernatant. Protein content was determined via Bio-Rad DC protein assay as per manufacturer's instructions (Bio-Rad Laboratories, NSW, Australia). Briefly, samples were separated by SDS-PAGE using Criterion™ TGX Stain-Free™ Precast Gels (Bio-Rad) and proteins were transferred to 0.45 mm PVDF via Trans-Blot® Turbo™ transfer system (Bio-Rad). Gels were visualized using a ChemiDoc™ imaging system (Bio-Rad) and images were captured prior to and following transfer. Membranes were blocked for 2 h at room temperature (RT) in 5% (w/v) bovine serum albumin (BSA, Sigma-Aldrich) or 5% Skim Milk (for puromycin) in Tris-buffered saline-Tween 20 (TBST). Membranes were incubated overnight at 4°C with primary antibodies. The following day membranes were incubated for 1 h at RT in horseradish peroxidise-conjugated secondary antibodies. Membranes were treated with enhanced chemiluminescence (Super Signal West Femto; Thermo Scientific) and proteins were visualized using ChemiDoc™ imaging system (Bio-Rad). Blots were quantified using ImageLab® software (Bio-Rad), and normalized to total protein as quantified from the Stain Free Gel imaging (**Figure 3**) or by BLOT FastStain™ as per manufacturer's instructions (G-Biosciences, St. Louis, MO; **Figure 2**).

#### Antibodies

pAkt (S473), Akt, pmTOR (S2448), mTOR, pp70S6K (Thr389), p70S6K, pS6 (S235/236), S6, p4EBP1 (T37/46), 4EBP1, pSTAT3 (Tyr705), and STAT3 were purchased from Cell Signaling Technologies (Beverly, MA, USA) and diluted 1:1,000 in 5% BSA/TBST. Secondary antibodies (donkey anti-rabbit HRP immunoglobulins, GE Healthcare Life Sciences, Australia) were diluted in 5% BSA/TBST.

To assess whether protein expression of key structural protein were affected by glycine in the wasting models, MF20 (Developmental Studies Hybridoma Bank) was diluted 1:50 in 5% BSA/TBST. α-actin (Sigma-Aldrich) was diluted 1/3,000 in 5% BSA/TBST. Secondary antibodies (sheep anti-mouse HRP immunoglobulins GE Healthcare Life Sciences) were diluted in 5% BSA/TBST.

Anti-puromycin (Clone 12D10; Millipore) was diluted 1:5,000 in 1% BSA/TBST. Secondary antibodies (goat anti-mouse IgG Fc 2a, Jackson ImmunoResearch Laboratories Inc., West Grove, PA, USA) were diluted 1:50,000 in 5% milk/TBST.

### Statistics

All values were expressed as mean ± SD. Data were normalized to the appropriate control group for ease of visualization, unless otherwise stated. Two-way ANOVAs (time and treatment) were used to compare between groups for dose-response, time-course, and rapamycin experiment, while one-way ANOVAs were used for all other comparisons. Tukey's *post hoc* test was used to determine significant differences between individual groups and *P* values reported in figures. For transparency, we report both significant differences (*P* < 0.05) and trends (*P* < 0.1) are reported where appropriate.

## Results

### Glycine Reduces Muscle Wasting in a Dose-Dependent Manner During Growth Factor Deprivation

**Figure 1:**
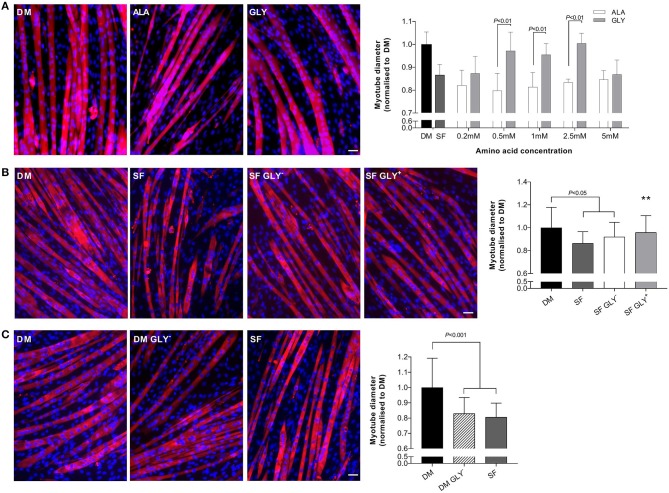
Glycine attenuates muscle wasting during growth factor deprivation. Myotube diameter for cells incubated in differentiation media (DM) or serum free (SF) media for 48 h with increasing concentrations of glycine or isomolar concentrations of L-alanine, and representative images at the optimal dose of 2.5 mM **(A)**. Removal of glycine from SF (SF GLY^−^) did not further impact myotube size compared to SF **(B)**. Removal of glycine from DM for 48 h induced myotube wasting like that in SF media **(C)**. Scale bar represents 50 μm. This applies to all images. Values are means ± SD, *n* = 4–6 per group. Significant differences are displayed where appropriate. ** Significantly different to SF, *P* < 0.01.

Growth factor deprivation (48 h) significantly reduced myotube diameter (SF, −13%; *P* < 0.05; Figure 1A). The addition of glycine to SF media attenuated muscle wasting in a dose-dependent manner, reaching significance at a concentration of 0.5, 1, and 2.5 mM. Importantly, equimolar concentrations of L-alanine did not attenuate myotube wasting, compared to SF alone. Serum starved glycine-treated myotubes were larger (0.5 mM, 18%; 1 mM, 16%; 2.5 mM, 22%; *P* < 0.01) compared with SF containing L-alanine-treated myotubes (SF ALA^+^). We have not further investigated why the beneficial effects of glycine were lost at a concentration of 5 mM as we used the dose-response studies to select the most appropriate and effective dose. All further experiments in SFM were performed using 2.5 mM glycine or L-alanine.

We also removed glycine from SF to determine if basal glycine contributed to any effects on myotube diameter. The removal of glycine (0.4 mM) from SF (SF GLY^−^) did not augment the effects of SF alone (Figure 1B).

### Glycine Is Important for Myotube Size Under Normal Growth Conditions

Removal of glycine from DM (48 h) induced myotube wasting similar to that caused by nutrient deprivation (SF). Differentiation media without basal glycine reduced myotube diameter by 17% compared to DM (*P* < 0.001; Figure 1C).

### Glycine Reduces Muscle Wasting in a Dose-Dependent Manner During Nutrient Starvation

**Figure 2:**
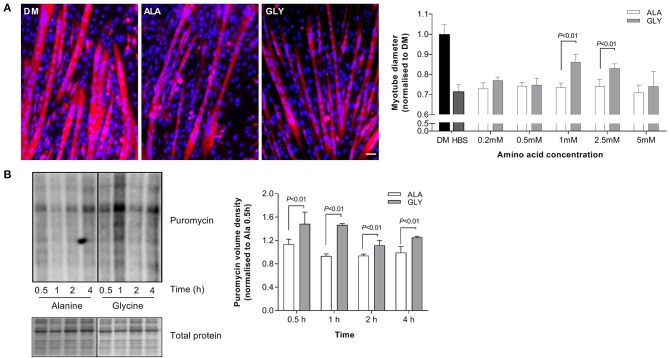
Glycine attenuates muscle wasting and preserves rates of protein synthesis during nutrient starvation. Myotube diameter for cells incubated in differentiation media (DM) or HEPES buffered saline (HBS) for 5 h with increasing concentrations of glycine or isomolar concentrations of L-alanine, and representative images at the optimal dose of 2.5 mM **(A)**. Glycine supplementation improves protein synthesis as assessed using puromycin after incubation in HBS for up to 4 h **(B)**. Scale bar represents 50 μm. This applies to all images. Values are means ± SD, *n* = 4–6 per group. Significant differences are displayed where appropriate.

Nutrient starvation (5 h) reduced myotube diameter (HBS, −29%; *P* < 0.05; Figure 2A). The addition of glycine to HBS attenuated muscle wasting in a dose-dependent manner, reaching significance at a concentration of 1 and 2.5 mM. Importantly, equimolar concentrations of L-alanine did not increase myotube diameter, compared to HBS alone. Nutrient deprived (HBS) glycine-treated myotubes were larger (1 mM, 18%; 2.5 mM 14%; *P* < 0.01) than myotubes treated with L-alanine (HBS ALA^+^). The beneficial effects of glycine were lost at a concentration of 5 mM. All further experiments in HBS were performed using 2.5 mM glycine or L-alanine.

### Glycine Maintains Protein Synthesis During Nutrient Starvation

Protein synthesis was improved by the presence of 2.5 mM glycine during 4 h of nutrient starvation. At each time point, puromycin incorporation was greater in glycine treated cells, compared to L-alanine treated cells (*P* < 0.01, Figure 2B).

### Glycine Protects From Muscle Wasting During Nutrient Starvation and Growth Factor Deprivation via mTORC1

**Figure 3:**
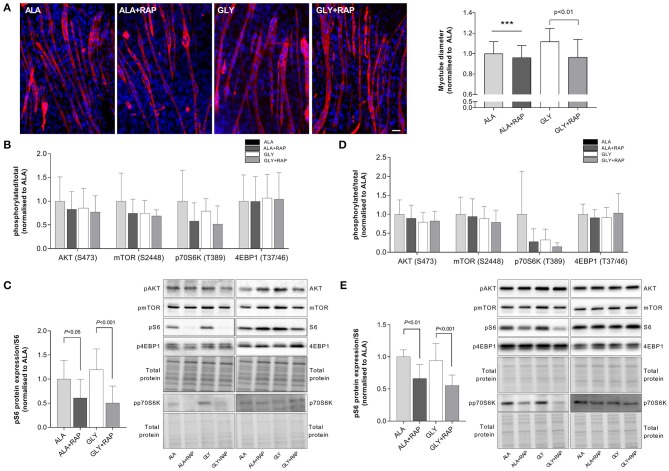
Glycine attenuates muscle wasting in an mTORC dependent manner. Representative images and myotube diameter for cells incubated in HBS for 5 h with 2.5 mM glycine or L-alanine co-treated with the mTORC1 inhibitor rapamycin (100 nM) **(A)**. Phosphorylation status of AKT, mTOR, p70S6K, 4EBP1 **(B)** and S6 **(C)** was measured following 4 h of HBS treatment with amino acids and rapamycin (100 nM). Phosphorylation status of AKT, mTOR, p70S6K, 4EBP1 **(D)**, and S6 **(E)** was measured following 4 h of SF treatment with amino acids and rapamycin (100 nM). Scale bar represents 50 μm. This applies to all images. Values are means ± SD, *n* = 4–8 per group. Significant differences are displayed where appropriate. *** Significantly different to GLY (treatment effect), *P* < 0.001.

Myotubes were incubated with the mTORC1 inhibitor rapamycin to determine the involvement of mTORC1 in the protective effect of glycine in the nutrient starvation and growth factor deprivation models. We measured the effects of rapamycin on myotube diameter and the phosphorylation status of downstream targets of mTORC1. In a model of 4 h nutrient starvation (HBS), rapamycin attenuated the protective effects of glycine by 13% (*P* < 0.01). There were no significant effects of rapamycin on myotube diameter in L-alanine treated cells (Figure 3A). Rapamycin also prevented the glycine-induced S6 phosphorylation (*P* < 0.001, Figure 3C). Similarly, when cells were treated acutely with amino acids and rapamycin in the growth factor deprivation model (4 h, SF), rapamycin prevented the maintenance of S6 phosphorylation (*P* < 0.001, Figure 3E). In L-alanine-treated cells, rapamycin also reduced S6 phosphorylation in both the deprivation (4 h HBS, *P* < 0.05) and starvation models (4 h SF, *P* < 0.01). No changes in AKT, mTOR, p70S6K or 4EBP1 phosphorylation were observed under any treatment condition (Figures 3B,D). Similarly, there were no changes in pSTAT3 phosphorylation or MF20 and α-actin protein abundance between the treatment groups (Supplementary Figure 1).

## Discussion

Calorie restriction is a common lifestyle intervention effective in combating obesity and the associated risk of metabolic diseases. Although glycine supplementation helps preserve lean mass in a mouse model of calorie restriction (5), the mechanisms for the protective effect have not been resolved. In the present study, we demonstrated that glycine protects against myotube wasting in an mTORC signaling-dependent manner in two *in vitro* models of nutrient/growth factor restriction.

Our model of nutrient starvation (HBS) has been shown previously to dramatically reduce protein synthesis and mTORC1 signaling and reduce myotube diameter (10). Similarly, 48 h of nutrient deprivation (SF) induced myotube atrophy. We observed a dose-dependent protection from myotube wasting in glycine treated C2C12 cells, in both HBS and SF models. The applied dose (2.5 mM) of glycine is ~10-fold higher than basal plasma concentrations, but only 40% higher than basal concentration in muscle and comparable to that seen after ingestion of a protein rich meal (12). Removal of glycine from SF media did not aggravate nutrient deprivation-induced atrophy. Interestingly, removal of glycine from differentiation media for 48 h induced atrophy, comparable to that of cells incubated in SF media. This may be attributed to a disruption in one-carbon metabolism by the loss of exogenous glycine availability and a subsequent inability to maintain cellular homeostasis (13, 14). The observation that glycine did not modulate STAT3 phosphorylation in HBS treated cells is in accordance with our previous studies demonstrating that glycine did not affect LPS-induced increases in cytokine production (6). For these reasons we did not pursue studying inflammatory pathways but focused instead on mTORC1 associated signaling.

In C2C12 myotubes incubated in HBS, glycine supplementation attenuated myotube wasting, maintained protein synthesis and promoted S6 phosphorylation. In both wasting models, the glycine-induced increases in S6 phosphorylation were completely prevented by rapamycin. Likewise, rapamycin inhibited the glycine-stimulated protection of myotube diameter, indicating that mTORC1 signaling is necessary for glycine's protective effects on myotube size in both growth factor deprivation and nutrient starvation models. These results are consistent with the effect of glycine on C2C12 myoblasts where acute exposure (30 min−6 h) promoted proliferation, cell viability and protein synthesis in the absence of serum, in an mTORC1-dependent manner (15). However, in myoblasts isolated from neonatal chicks, there was no effect of glycine supplementation on mTOR expression (16). The meaning of these observations is unclear since this study measured mTOR and S6K1 mRNA expression rather than the phosphorylation status of these signaling molecules as a measure of mTOR activity. Importantly for the present study, although rapamycin treatment reduced myotube diameter and S6 phosphorylation in L-alanine and glycine treated myotubes, L-alanine did not provide protection from atrophy during wasting conditions.

Upon activation, mTORC1 phosphorylates and activates two parallel signaling pathways. S6 kinase 1 phosphorylation leads to activation of the ribosomal protein S6, while phosphorylation of the eukaryotic initiation factor 4E (eIF4E)-binding protein (4EBP1) ultimately allows for the synthesis of new proteins. Despite inhibition of S6 phosphorylation by rapamycin in glycine treated cells, there was no alteration in phosphorylation of mTOR, P70S6K and 4EBP1 in either of the nutritional wasting models after 4 h of treatment suggesting that upstream signaling occurs earlier. Divergence in signaling between S6 and 4EBP1 has been described previously in glycine treated C2C12 myoblasts (15), and myotubes treated with other amino acids (10) and the reason for these discrepancies remain to be established.

The way in which many amino acids signal to mTORC1 is still relatively unknown. It is possible glycine may signal to mTORC1 via a non-canonical mechanism using Vps34 (17–19). However, it remains to be established whether glycine can mediate Vps34 expression in skeletal muscle. Alternatively, glycine may signal to mTORC1 via its receptor, GlyR (7). GlyRs are inhibitory Cl^−^ channels composed of three different subunits: (1) the ligand binding α subunits (GLRA1, GLRA2, GLRA3, or GLRA4); (2) a structural β subunit (GLRB); and (3) a cytoplasmic anchoring protein known as gephyrin (GPHN). GlyR mediated cell-cell communication is enabled by gephyrin, an anchoring protein that provides the scaffolding needed for cytoplasmic GlyRβ binding (20). Interestingly, it has been suggested gephyrin is required for mTORC1 signaling and contributes to the intracellular localization of mTORC1 and downstream signaling in a variety of cell types (8, 21). Although a functional GlyR is still to be identified in skeletal muscle, its presence in cardiac and smooth muscle suggests it is highly likely (22–24). Further studies are required to confirm GlyR-mTORC signaling in skeletal muscle.

In summary our results in C2C12 myotubes demonstrate a role for glycine in the protection of skeletal muscle wasting *in vitro*. Rapamycin inhibited the glycine-stimulated protection of myotube diameter, and the glycine-stimulated S6 phosphorylation, indicating that mTORC1 signaling is necessary for glycine's protective effects *in vitro*. The removal of glycine from differentiation media for 48 h highlights that glycine is required to maintain cellular homeostasis under normal differentiation conditions. Our results suggest that increasing glycine availability may be beneficial for muscle wasting conditions associated with inadequate nutrient intake.

## Data Availability Statement

The datasets generated for this study are available on request to the corresponding author.

## Author Contributions

MC, DH, GL, and RK interpreted results, conception and design of research, edited and revised manuscript, and approved final version of manuscript. MC, DH, JC, and JT performed experiments. MC and DH analyzed data. MC drafted manuscript.

### Conflict of Interest

The authors declare that the research was conducted in the absence of any commercial or financial relationships that could be construed as a potential conflict of interest.

## Supplementary Material

Supplementary Figure 1 Phosphorylation of STAT3 and MF20 and α-actin protein abundance was measured following 4 h of HBS **(A)** or SFM **(B)** treatment with amino acids and rapamycin (100 nM). Values are means ± SD, *n* = 4 per group.
