# Supplementary figures and images for: Glycine Protects Muscle Cells From Wasting in vitro via mTORC1 Signaling
Source: Front Nutr. 2019 Nov 13;6:172. doi: 10.3389/fnut.2019.00172 (PMC6871541; doi:10.3389/fnut.2019.00172)

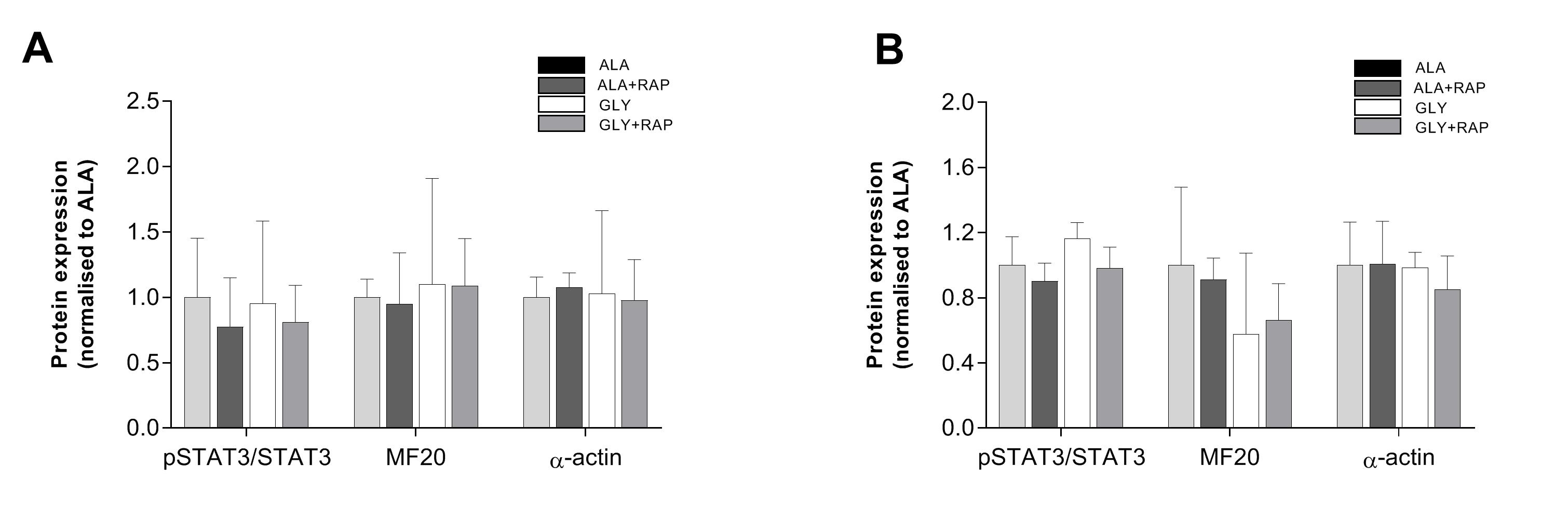

Supplement: Supplementary Figure 1 — Phosphorylation of STAT3 and MF20 and α-actin protein abundance was measured following 4 h of HBS (A) or SFM (B) treatment with amino acids and rapamycin (100 nM). Values are means ± SD, n = 4 per group. [file Image_1.JPEG]
